# Peer review of "Impact of LEAP and CBT-AN Therapy on Improving Outcomes in Women with Anorexia Nervosa"

_behavsci, 2023, doi:10.3390/bs13080651_

Round 1

Reviewer 1 Report

In the manuscript „Impact of LEAP and CBT-AN Therapy on improving outcomes in women with anorexia nervosa.“ Hay et al. report about the comparison between the effects of compuLsive Exercise Activity TheraPy vs. cognitive behavioral therapy for AN on numerous survey measures and BMI. Although the study is very comprehensive the following points need to be addressed:

Major:

1.                  There is a high risk for a selection bias and limited generalizability due to the low number of patients included (78) from a cohort of 574 patients in total, with an additional loss to follow-up of 35 patients. Please reconsider further recruitment of patients.

2.                  The levels of significance need to be adjusted based on multiple testing for multiple endpoints to reduce the risk of type 1 error.

3.                  The statement „The effect sizes for baseline to end of therapy assessment showed that effect sizes for LEAP were slightly larger than for the CBT-AN group for most of the measures“ in the results and discussion appears misleading since no statistical testing of the difference was performed. The absolute differences could be random and not significant.

4.                  It would be of special interest to identify factors (certain cut-offs in survey measures or sociodemographic measures) that are associated with a significantly better outcome with LEAP or CBT-AN, to be able to offer a more individualized therapy for AN patients and identify those that would benefit the most from LEAP.

5.                  The discussion should go more into a detail when comparing the novel findings with knowledge from literature so far.

Minor:

1.                  In the title the word „therapy“ should not start with a capital letter. Moreover, The use of the abbreviation „LEAP“ in the title should be re-evaluated, since it can be confused with the liver-enriched antimicrobial peptide. Overall the term „LEAP therapy“ is a repetition since the P of “LEAP“ indicates theraPy.

2.                  The word „therapy“ in the manuscript should not start with a capital letter. Additionally, there are several mistakes regarding the use of capital letters in the manuscript.

3.                  Please report specific numeric values as well as p-values in the abstract.

4.                  Specify „most other therapies“ in ll.64.

5.                  Some references need revision: Add a reference to ll.73. Revise the reference in ll.95. Mention the reference 22 in the paragraph from ll.104-110 after every sentence.

6.                  Regarding table 1 please add a statistical comparison between LEAP and CBT-AN group for all factors (all ages, all education levels, all employment types etc.).

7.                  In table 2 there are no ↓ab or ↑b as described in the note section of the table.

8.                  Please revise suppl. File 1, the arrows are hard to understand.

This study is comprehensive with many very interesting results, however the statistical analysis needs improvement by adjusting the significance level for multiple testing and performing additional tests to examine the difference in effect sizes and identify the patients most suitable for LEAP by regression analysis.

few typs - please correct

Author Response

Reviewers Comments and Reply 

Reviewer -1

Although the study is very comprehensive the following points need to be addressed:

Major

  1. There is a high risk for a selection bias and limited generalizability due to the low number of patients included (78) from a cohort of 574 patients in total, with an additional loss to follow-up of 35 patients. Please reconsider further recruitment of patients.

Our response: Thanks for your suggestions about to “reconsider further recruitment of patients”. It is be to be noted that this study has already completed the treatment intervention and post-treatment 6-month follow-up assessment completed in 2016. A brief paper has already been published (Hay et al. 2018) and this is secondary analysis of the data which is indicated in methods section. There is no scope for further recruitment of patients. However, we addressed this small sample size issue and loss of follow-up in discussion section as one of the main limitations of this study (lines 509-605).  “One of the main limitations of this study that during the follow-up period 35.1% (26 of 74) participants lost follow-up between baseline assessment and end of Therapy assessment; and overall, 44.6% (33 of 74) lost follow-up between baseline and 6-months follow-up assessment. Participants who were lost to follow-up between baseline and 6-months assessment periods were comparatively younger but not significantly than those retained in the sample (mean age at baseline: 26.2 years vs. 27.7 years; p=0.46. The differences in their reported levels of education (p=0.67), employment status (p=0.55) and psychotropic medication use (p=0.98) at baseline were not found to be statistically significant between two groups (Appendix C). A similar non-significant difference was also observed in baseline outcome measures including mean body mass index (p=0.84), SF12MCS (p=0.15), EDQoL (p=0.10), weight concern (p=0.05), dietary restraint (p=0.17), eating concern (p=0.18), compulsive exercise test (p=0.87) and exercise beliefs questionnaire (p=0.37 (Appendix C). This finding indicates that despite loss of 44.6% subjects at 6-months follow-up which assumed to be occurred at random, the loss of participants at follow-up is unlikely to have impacted on the overall findings of this study. However, we have advised caution in  in generalizing our results prior to more research.”

  1. The levels of significance need to be adjusted based on multiple testing for multiple endpoints to reduce the risk of type 1 error.

 Our response: Thank you for this comment. In addition to multiple comparison test we now added the results from mixed modelling (new Table 2), mixed model is adjusted for both treatment group and multiple assessment points. It is to be noted that in multiple comparison test we applied Bonferroni statistical test to examine pairwise significant differences across time points (T0, T1, T2) within each treatment group. Accordingly, the methods and results section also revised. Both mixed modelling and multiple comparison test showed significant differences across time points and majority p-values are <0.01. In revised discussion section we mentioned due to small sample size the interpretation of results needs additional cautious. In methods we revised text (lines 282-295): “As the outcome measures are continuous, we applied mixed models to examine the adjusted interaction effects for time (three assessment periods: baseline, end of therapy and six-months follow-up) and group (LEAP, CB-TN) effects. The method allows flexibility in modelling covariance structures involving longitudinal and repeated data of a correlated type, considering within-subject, time dependent correlations. In addition to this multiple group comparison test between ‘baseline (T0) vs. end of therapy (T1) assessment’; baseline vs. 6-month follow-up (T2) assessment’; and ‘T1 vs. T2’ were conducted through statistical technique ANOVA (analysis of variance) for repeated measures. Bonferroni statistical test was applied to examine the pairwise significant differences across time points (that is, T0 vs. T1; T0 vs. T2; and T1 vs. T2) for all outcome measures controlling for LEAP and CBT-AN participants.”

  1. The statement „The effect sizes for baseline to end of therapy assessment showed that effect sizes for LEAP were slightly larger than for the CBT-AN group for most of the measures“ in the results and discussion appears misleading since no statistical testing of the difference was performed. The absolute differences could be random and not significant.

Our response: Thank you for this comment. Too examine the significant differences of effect sizes between LEAP and CBT-AN group we now added 95%CIs for all estimated effect sizes presented in Table 4. Accordingly, we also added 95%CIs of effect sizes in result section.

  1. It would be of special interest to identify factors (certain cut-offs in survey measures or sociodemographic measures) that are associated with a significantly better outcome with LEAP or CBT-AN, to be able to offer a more individualized therapy for AN patients and identify those that would benefit the most from LEAP.

Our response:  As per suggestions, we now added a new Table 4a which presents the effect sizes with 95%CIs between baseline and 6-month follow-up assessment for four main outcome measures i.e., including body mass index, EDE-Q Global score, SF12-MCS and EDQoL by socio-demographic characteristics for LEAP and CBT-AN participants respectively.

  1. The discussion should go more into a detail when comparing the novel findings with knowledge from literature so far.

Our response: In discussion section we now included more details about the novel findings with knowledge from literature.

Please see text in discussion (lines 572-588): “The present study has implications for the treatment for AN. The findings support the body of literature that, and in contrast to other interventions such as medications, endorse psychological therapies for AN showing continued improvements in symptoms over time, albeit with smaller effect sizes than during the active phase of therapy [5]. Further, the results indicate that several baseline characteristics, BMI, level of ED symptoms (EDE-Q Global score) and MHRQoL are consistent with previous literature [32]. To our knowledge the additional finding that these characteristics at end of treatment also predicted 6-month outcomes is novel. Thus, assessment of these features is important not only for identifying whether a treatment is likely to be effective, but also that these effects will be sustained over time. Whilst not being predictive two measures: psychological distress and AN stage of motivation to change, were found to be significantly associated with ED symptoms and EDQoL; and again this is consistent with the broader eating disorders research [32]. Future treatment programs should aim to reduce psychological distress and increase motivation during CBT-AN treatment because although it may not directly predict change in psychopathology at the end of therapy, participants will be in a better place for eventual recovery.”

Minor:

  1. In the title the word „therapy“ should not start with a capital letter. Moreover, The use of the abbreviation „LEAP“ in the title should be re-evaluated, since it can be confused with the liver-enriched antimicrobial peptide. Overall the term „LEAP therapy“ is a repetition since the P of “LEAP“ indicates theraPy.

Our response:  We now replaced title word ‘Therapy’ by ‘therapy’. As per comments, throughout the manuscript we also deleted the word ‘therapy’ from ‘LEAP therapy“.   

  1. The word „therapy“ in the manuscript should not start with a capital letter. Additionally, there are several mistakes regarding the use of capital letters in the manuscript.

Our response:  We now replaced title word ‘Therapy’ by ‘therapy’. As per comments, throughout the manuscript we also corrected the typos related to the use of capital letters.   

  1. Please report specific numeric values as well as p-values in the abstract.

Our response:  As there are too many outcomes, due to word limitations in abstract - numerical and p-values are not included. However, p-values are included Tables and results section.

  1. specify „most other therapies“ in ll.64.

Our response:  We now included other specified therapies (e.g., FT-AN, family based treatment for anorexia nervosa; IPT, interpersonal psycho-therapy; SSCM, specialist supportive clinical management; CRT, cognitive remediation therapy; CAT, cognitive-analytic therapy),

  1. Some references need revision: Add a reference to ll.73. Revise the reference in ll.95. Mention the reference 22 in the paragraph from ll.104-110 after every sentence.

Our response:   As per suggestion a reference [11] added to  ll.73 (new 74)[Dittmer, N., C. Jacobi, and U. Voderholzer, Compulsive exercise in eating disorders: proposal for a definition and a clinical assessment. J Eat Disord, 2018. 6: p. 42.]. Refence in ll.95 revised as [12].  Reference included after every sentence in the specific paragraph.

  1. Regarding table 1 please add a statistical comparison between LEAP and CBT-AN group for all factors (all ages, all education levels, all employment types etc.).

Our response:  As per suggestion, in Table 1 we added p-values from statistical comparison tests to examine differences between LEAP and CBT-AN group for all factors.

  1. 7. In table 2 there are no ↓ab or ↑b as described in the note section of the table.

Our response: These typos are corrected in the revised manuscript (new Table 3).

  1. Please revise suppl. File 1, the arrows are hard to understand.

Our response:  To better understand the suppl. File 1, we now simplified flowchart and presented as an image file.

Reviewer 2 Report

This study appears to be a secondary report examining data from an RCT of 34 sessions of CBT-AN vs 26 sessions of CBT-AN + 8 sessions on exercise and motivation for change (LEAP) and included 74 participants (38 CBT-AN and 36 LEAP) across 3 international sites (UK, US, Australia). Participants were assessed at baseline, EOT and 6-mo followup.

The main aims listed are to 1) examine baseline predictors of outcome and 2) estimate magnitude of change for outcome measures controlling for group 3) explore correlations between outcome measures at each of the 3 time points and 4) examine association of main outcome measures with baseline participant characteristics and secondary outcomes.

Suggest making more explicit in introduction on p 3 that this is a secondary analysis of a previously published study at least I think that is what I understand. Participant n’s seem to vary slightly between the papers (Hay et al., 2018 and this submission) -- reason for this is unclear e.g, numbers by site are higher in some cases lower in others for the two reports which looks odd.

The paper and analyses are challenging to follow and would benefit from significant editing and focus. The outcome variables and aims should be articulated clearly and the analyses are confusing. For example:

P 9 (results section) talks about primary and secondary outcome measure results however what is a primary vs secondary outcome variable is not defined in introduction aims or in methods sections and seem to vary throughout by analysis. On line 285 primary outcomes are listed as BMI, EDEQ global, SF12MCS and EDQoL but later in Table 2 primary outcome measures are BMI and EDEQ scores (global and subscales) with Table 2a listing all other instruments as secondary outcome measures. Which is it? Later in the correlation analysis it appears that perhaps secondary measures are ANSOCQ, EBQ, CET, K10 and EDEQ subscores?

Mixing up questionnaires and variables they purport to assess is confusing for the reader– suggest sticking with listing questionnaire acronyms eg line 329 mentions psychological distress rather than Kessler-10, together with SF12MCS and SF12PCS which elsewhere are referred to as the P/MHRQoL measures. Same line includes AN stage of change which elsewhere is more clearly labelled as the ANSOCQ score. Recommend checking consistent use of all acronyms once introduced. Same goes for the EBQ. This is also the case in tables eg table 2a some questionnaires are referred to by acronym others by name. Given the number of instruments used this gets confusing to follow.

Table 2 – there are different type “a’s” and foot note refers to “ab” and “b” superscripts neither of which appear in table at all. Appendix B table has similar problem with footnotes. Would avoid using two fonts of letter “a” as different superscripts in multiple tables.

Unclear if same therapists did both interventions in the description given in limitations section of discussion on p23 or why this appears here at all when not included in the methods section (I realize reader can find it in the prior article by the group - maybe a reference to this would be helpful – no need to duplicate otherwise)

There appears to be no effect for LEAP on BMI at EOT yet this is not mentioned. Given weight restoration remains the best predictor of outcome for AN this should be in abstract, or minimally explicitly discussed in discussion section as well as in the comments on appendix B (line 375). I realize that at follow up there appears to be no group difference but it is notably absent at EOT.

Other

A close read is advised as there are numerous typos throughout as well as grammatical issues

Some examples include:

Line 124, 298, 441,443. Lines 543-545. Line 554-557, 558, 560, 564

Needs careful editing. Many typos and several non grammatical paragraphs

Author Response

Reviewer -2

Comments and Suggestions for Authors

#1. Suggest making more explicit in introduction on p 3 that this is a secondary analysis of a previously published study at least I think that is what I understand. Participant n’s seem to vary slightly between the papers (Hay et al., 2018 and this submission) -- reason for this is unclear e.g, numbers by site are higher in some cases lower in others for the two reports which looks odd.

Our response:  We revised the introduction by indicating that this the secondary analysis. Regarding "Participant n’s seem to vary slightly between the papers (Hay et al., 2018 and this submission): It is to be noted that in the original study there were 78 participants included at baseline, among them only 4 were male (5.1%) and 74 were female (94.9%). The disproportionate number of males may introduce gender bias in the estimated results and the results may not be generalizable to overall population level. Considering gender bias in the sample, for this study only 74 female participants are included in the final analytic sample (Supplementary File 1). This has been mentioned in 2.1 Study Design and Participants, Paragraph 2.

#2 The paper and analyses are challenging to follow and would benefit from significant editing and focus. The outcome variables and aims should be articulated clearly and the analyses are confusing. For example: P 9 (results section) talks about primary and secondary outcome measure results however what is a primary vs secondary outcome variable is not defined in introduction aims or in methods sections and seem to vary throughout by analysis. On line 285 primary outcomes are listed as BMI, EDEQ global, SF12MCS and EDQoL but later in Table 2 primary outcome measures are BMI and EDEQ scores (global and subscales) with Table 2a listing all other instruments as secondary outcome measures. Which is it? Later in the correlation analysis it appears that perhaps secondary measures are ANSOCQ, EBQ, CET, K10 and EDEQ sub scores?

Our response:  We now clearly indicated the primary and secondary measures in methods section. Accordingly corrected the typos throughout the manuscript.

#3. Mixing up questionnaires and variables they purport to assess is confusing for the reader– suggest sticking with listing questionnaire acronyms eg line 329 mentions psychological distress rather than Kessler-10, together with SF12MCS and SF12PCS which elsewhere are referred to as the P/MHRQoL measures. Same line includes AN stage of change which elsewhere is more clearly labelled as the ANSOCQ score. Recommend checking consistent use of all acronyms once introduced. Same goes for the EBQ. This is also the case in tables eg table 2a some questionnaires are referred to by acronym others by name. Given the number of instruments used this gets confusing to follow.

Our response:  As per suggestion we checked the consistency of the use of acronyms throughout the manuscript and corrected accordingly.

#4. Table 2 – there are different type “a’s” and foot note refers to “ab” and “b” superscripts neither of which appear in table at all. Appendix B table has similar problem with footnotes. Would avoid using two fonts of letter “a” as different superscripts in multiple tables.

Our response:  These typos are corrected in the revised manuscript.

#5. Unclear if same therapists did both interventions in the description given in limitations section of discussion on p23 or why this appears here at all when not included in the methods section (I realize reader can find it in the prior article by the group - maybe a reference to this would be helpful – no need to duplicate otherwise)

 Our response:  As per comment, we have edited this for clarity (lines 169-170 in methods and line 619-21 in the discussion) and individual therapists were trained in and provided both therapies.

 #6. There appears to be no effect for LEAP on BMI at EOT yet this is not mentioned. Given weight restoration remains the best predictor of outcome for AN this should be in abstract, or minimally explicitly discussed in discussion section as well as in the comments on appendix B (line 375). I realize that at follow up there appears to be no group difference but it is notably absent at EOT.

 Our response:  The effect for LEAP and CBT-AN on BMI at the end of therapy are included revised manuscript. The effect of weight concern and AN stage of change are already included in Abstract. Due to word limitations the Abstract could not accommodate many important findings.  

#7. A close read is advised as there are numerous typos throughout as well as grammatical issues

Some examples include: Line 124, 298, 441,443. Lines 543-545. Line 554-557, 558, 560, 564

Our response:  Grammatical error in above indicated text and as well other part of the manuscript has been corrected.

Round 2

Reviewer 1 Report

The authors sufficiently addressed most of the comments; however, three remain:

Major comments:

1.       The first major comment regarding the small sample size was not addressed at all. The authors need at least to discuss the high risk for a selection bias and limited generalizability and add the small number of patients to their limitations.

2.       Since in response to the second major comment “Bonferroni statistical test was applied to examine the pairwise significant differences across time points”, the adjusted significance levels should be indicated with the results.

Minor comment:

1.       Please revise suppl. File 1, the arrows appear chaotic.

Author Response

Reviewer -1

The authors sufficiently addressed most of the comments; however, three remain: 

Major comments:  

#1. The first major comment regarding the small sample size was not addressed at all. The authors need at least to discuss the high risk for a selection bias and limited generalizability and add the small number of patients to their limitations.  

Our response: Thanks for your suggestions. We now addressed the small sample size issues, selection bias and limited generalizability as potential limitations in discussion section (new lines 632-644). 

“A significant limitation of this study is that there was a possible sampling bias in the recruitment from ED clinics and through community advertisements in three cities of three different countries. Due to the small number of participants overall and particularly from each country (UK: 38, Australia: 23, USA: 13) all the analyses were carried out on a combined sample which may not be representative of people receiving treatment for anorexia nervosa for each country or other countries. As with RCTs where there are strict inclusion criteria, results also may not be generalizable to all who have a need for treatment as this would include people with e.g., a diagnosis of bipolar disorder or current substance dependence who were excluded from the present RCT. These study features limit the generalizability regarding the findings. Furthermore, the majority of data used for statistical analyses in this study were obtained using self-report measures. It is likely that more accurate clinical data could have been obtained by using semi-structured interviews where the interviewer can explore symptoms experienced by participants in depth [46].”

 #2.  Since in response to the second major comment “Bonferroni statistical test was applied to examine the pairwise significant differences across time points”, the adjusted significance levels should be indicated with the results.  

Our response: The raw and adjusted p-values from Bonferroni statistical test are presented in Appendix Table A. As per comments interpretation of the results are also included in text (new lines 403-23).

“Appendix A reports the pairwise significant mean differences (raw and adjusted p-values) across different time points (i.e., T0 vs. T1; T0 vs. T2; and T1 vs. T2) through the Bonferroni statistical test controlling for the LEAP and CBT-AN group participants. Each matched sample comprised all participants who completed each outcome measure between the two time points under comparison. The results in Appendix A showed a similar pattern as presented in Table 3. Both LEAP and CBT-AN participants reported significantly lower scores on all mental health indices between T0 and T1, and T0 and T2 (all p <0.05). It is to be noted that between T0 and T1, and T0 and T2, based on raw (unadjusted) p-values 85% of findings were significant at p<0.01, and applying adjustments of p-values 81% findings become significant at p<0.01. As shown on the table in Appendix A, the mean BMI score for both groups increased from T0 to T1 and was found to be statistically significant among CBT-AN participants only (p <0.001) but was not found to be statistically significant among LEAP participants. The mean score of exercise beliefs between T0 and T1 for participants of both intervention groups had overall p=0.001 but for CBT-AN participants was p=0.03 (adjusted p=0.09). Furthermore, the mean scores of all the mental health measures significantly differed between T0 and T2 except for exercise beliefs within each intervention group and SF12MCS among LEAP participants, but combined results for participants in both intervention groups reached significance (p=0.015 for exercise beliefs and p=0.001 for SF12MCS). Between T1 and T2 there were no significant differences for any of the outcome measures except for dietary restraint among the CBT-AN group (p =0.016, adjusted p=0.048), this might be because of shorter follow-up time duration.” 

Minor comment:  

#1.  Please revise suppl. File 1, the arrows appear chaotic.  

 Our response: As per suggestion, we now revised the arrows. 

Reviewer 2 Report

The authors have not addressed this reviewer's prior comments adequately.

Numerous, including new, typos and inconsistencies remain (for example what is CB-TN? and why is there a down arrow in table 3 for both types of "a"s ?  Line 542 reads "...might have inexperienced symptomatic worsening". Use of acronyms continues to be inconsistent e.g. why is the ANSOCQ acronym selectively not utilized?

Main outcome measures continue to vary throughout manuscript. EDQoL for example is called a primary outcome measure in some places 9e.g. line 300) and is called as a secondary one in others (line 359)

Sections would benefit from being more concise and clear eg lines 114-138 of introduction should be rewritten to focus the aims of the paper for the reader. In general the paper would benefit from significant editing.

Author Response

Reviewer – 2

The authors have not addressed this reviewer's prior comments adequately. 

#1. Numerous, including new, typos and inconsistencies remain (for example what is CB-TN? and why is there a down arrow in table 3 for both types of "a"s ?  Line 542 reads "...might have inexperienced symptomatic worsening". Use of acronyms continues to be inconsistent e.g. why is the ANSOCQ acronym selectively not utilized? 

Our response: We now corrected the typos throughout the manuscript. 

Regarding arrows in Table 3 – we now treated ‘down arrow (↓a)’ with superscript ‘a’; and ‘up arrow (↑b)’ with superscript ‘b’. ↓a Indicates that mean score is significantly (p<0.05) lower than baseline (T0) score; and (↑b) Indicates that mean score is significantly (p<0.05) higher than baseline (T0) score.

Line 542 now 592 reads "...might have inexperienced symptomatic worsening". We now corrected typos in the sentence. “… . might have experienced symptomatic worsening”.  

As per comments, instead of ’AN stage of change’ we now used the acronym ‘ANSOCQ’ throughout manuscript. 

#2. Main outcome measures continue to vary throughout manuscript. EDQoL for example is called a primary outcome measure in some places 9e.g. line 300) and is called as a secondary one in others (line 359). 

Our response: As per comments we now deleted primary and secondary words. Instead of primary and secondary we used all outcome measures. For analytical purposes we treated body mass index, EDE-Q Global score, SF12MCS, EDQoL as main outcome measures. 

#3. Comments on the Quality of English Language 

Sections would benefit from being more concise and clear eg lines 114-138 of introduction should be rewritten to focus the aims of the paper for the reader. In general the paper would benefit from significant editing. 

 Our response: As per suggestion above indicated lines (114-138) of introduction has been rewritten, and whole manuscript has been edited.

 “This is secondary detailed analysis of the original data from an RCT of CBT-AN and LEAP that has been previously published as a brief paper [23]. The purpose of this study was to extend others’ findings and examine factors associated with out-comes in a RCT of CBT-AN and LEAP prior to therapy (baseline), at the end of therapy and at 6-month follow-up after therapy. Whilst specific ED symptoms, such as eating concern [27] and shape concern [28] have found to be predict poorer treatment outcomes [29, 30] variability in the definition and measurement of ED features such as BMI, ED psychopathology and EDQoL [31] and the focus of the literature on pre-treatment baseline predictors [32] has resulted in inconsistent findings and limited interpretations of previous research. Thus, the main aim of this study was to explore putative predictors of outcome (namely, BMI, ED symptoms, readiness to change, psychological distress, EDQoL and general health related quality of life) and to estimate the magnitude of their changes between baseline to end of therapy; base-line to 6-month follow-up; and end of therapy to 6-month follow-up. Finally, the as-sociation of specific outcome measures (BMI, EDE-Q Global score, EDQoL, and SF12MCS) with participant characteristics was also examined.”

Round 3

Reviewer 1 Report

The authors sufficiently addressed all comments.

Author Response

Thanks for reviewing our manuscript. Based on your review the quality of our manuscript has improved a lot.