# Supplementary material for: Impact of LEAP and CBT-AN Therapy on Improving Outcomes in Women with Anorexia Nervosa
Source: Behav Sci (Basel). 2023 Aug 3;13(8):651. doi: 10.3390/bs13080651 (PMC10451721; doi:10.3390/bs13080651)

Supplementary Figure S1. Participant flowchart covering eligibility of participants, baseline assessment, end of therapy assessment and 6-month follow-up assessment.

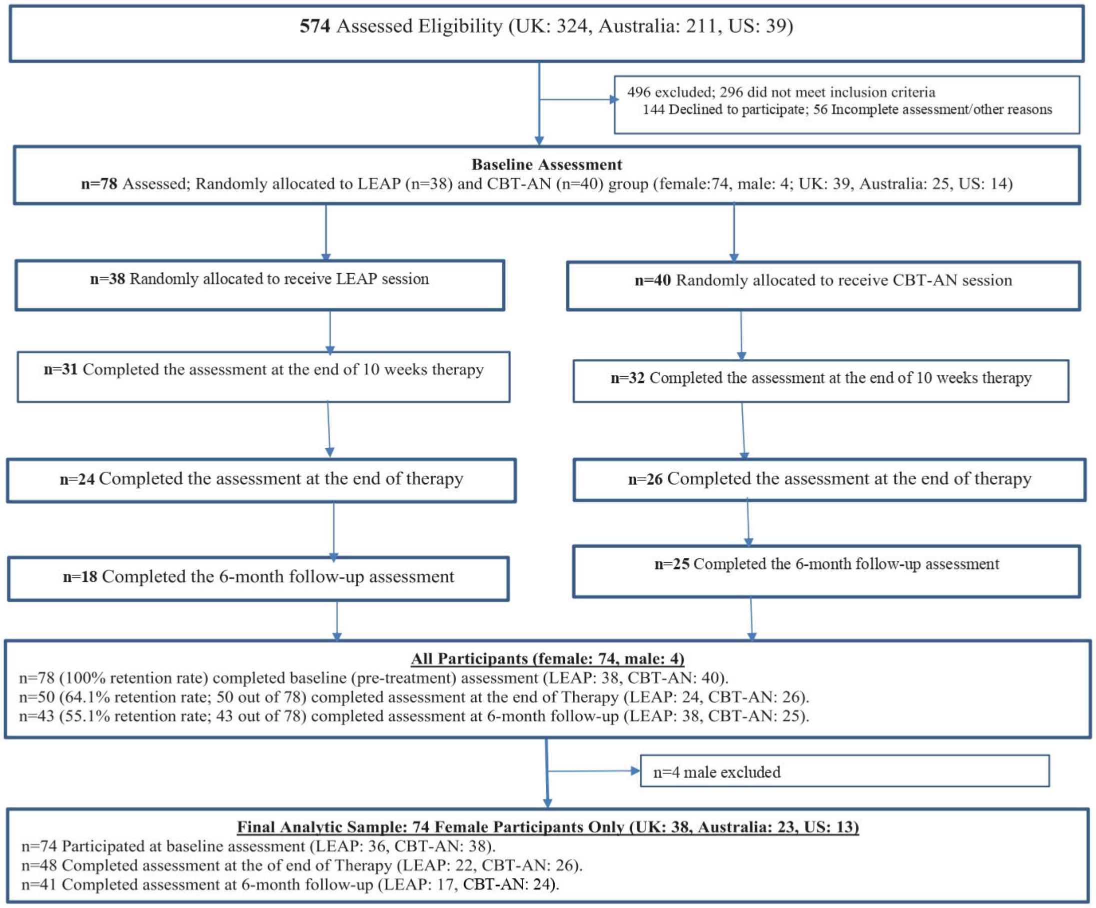

Supplement: Supplementary file 1 [file behavsci-13-00651-s001.zip › behavsci-2442383-supplementary.pdf]
